# IRFNet: Cognitive-Inspired Iterative Refinement Fusion Network for Camouflaged Object Detection

**DOI:** 10.3390/s25051555

**Published:** 2025-03-03

**Authors:** Guohan Li, Jingxin Wang, Jianming Wei, Zhengyi Xu

**Affiliations:** 1 Shanghai Advanced Research Institute, Chinese Academy of Sciences, Shanghai 201210, China; ligh@sari.ac.cn (G.L.); wangjx2022@shanghaitech.edu.cn (J.W.); 2 School of Electronic, Electrical and Communication Engineering, University of Chinese Academy of Sciences, Beijing 100049, China; 3 School of Information Science and Technology, ShanghaiTech University, Shanghai 201210, China

**Keywords:** object detection, computer vision, camouflaged object detection, attention mechanism, iterative refinement, cross-level feature fusion

## Abstract

Camouflaged Object Detection (COD) aims to identify objects that are intentionally concealed within their surroundings through appearance, texture, or pattern adaptations. Despite recent advances, extreme object–background similarity causes existing methods struggle with accurately capturing discriminative features and effectively modeling multiscale patterns while preserving fine details. To address these challenges, we propose Iterative Refinement Fusion Network (IRFNet), a novel framework that mimics human visual cognition through progressive feature enhancement and iterative optimization. Our approach incorporates the following: (1) a Hierarchical Feature Enhancement Module (HFEM) coupled with a dynamic channel-spatial attention mechanism, which enriches multiscale feature representations through bilateral and trilateral fusion pathways; and (2) a Context-guided Iterative Optimization Framework (CIOF) that combines transformer-based global context modeling with iterative refinement through dual-branch supervision. Extensive experiments on three challenging benchmark datasets (CAMO, COD10K, and NC4K) demonstrate that IRFNet consistently outperforms fourteen state-of-the-art methods, achieving improvements of 0.9–13.7% across key metrics. Comprehensive ablation studies validate the effectiveness of each proposed component and demonstrate how our iterative refinement strategy enables progressive improvement in detection accuracy.

## 1. Introduction

Camouflage is a widespread phenomenon in nature by which organisms evolve to blend with their surroundings through adaptation of appearance, coloration, or pattern [1,2,3]. This biological defense mechanism has evolved in response to harsh living environments, as exemplified by chameleons, cuttlefish, and flatfish. Camouflaged Object Detection (COD) aims to identify such concealed objects from their surroundings [4]. COD presents unique challenges compared to both traditional object detection tasks [5,6] and Salient Object Detection (SOD) [7,8,9], where targets typically stand out from their environment. Recently, COD has garnered increasing research interest due to its wide applicability in medical analysis (e.g., polyp segmentation [10,11,12]), agriculture [13], and industrial detection tasks [14].

The inherent challenges of COD stem from several factors: the extreme similarity between objects and backgrounds makes feature discrimination exceptionally difficult; in addition, camouflaged objects vary dramatically in size, shape, and appearance, ranging from tiny insects against complex textures to larger animals with disruptive coloration patterns. These objects often have boundaries that seamlessly blend with environmental elements, while partial occlusions and complex backgrounds further complicate accurate detection and segmentation.

Early COD approaches relied primarily on handcrafted features such as color, texture, and intensity; however, these traditional methods often struggled with complex scenes due to their limited semantic understanding capabilities. With the advent of deep learning, more advanced approaches have emerged, demonstrating superior detection accuracy and generalization ability. Nevertheless, existing deep learning-based methods still face limitations in distinguishing fine-grained features and perceiving subtle differences between objects and their surroundings, frequently producing incomplete or imprecise results when confronted with well-camouflaged targets.

When observing camouflaged objects in natural settings, the human visual system employs a sophisticated process of perception that relies on two key capabilities. First, highly discriminative feature perception allows humans to notice subtle discontinuities or peculiar patterns that reveal a camouflaged animal. Second, iterative observation refinement enables progressive analysis where initial observations might only capture partial features, which then activate prior knowledge to formulate recognition hypotheses. These hypotheses are progressively refined by focusing attention on potentially discriminative features until successful recognition occurs. Inspired by these cognitive processes, we propose an Iterative Refinement Fusion Network (IRFNet) that mimics such perceptual mechanisms. To enhance discriminative feature perception, we design a Hierarchical Feature Enhancement Module (HFEM) that employs bilateral and trilateral fusion pathways to establish cross-scale feature connections, complemented by a dynamic channel-spatial attention mechanism that adaptively emphasizes the most informative features in a similar way to how human attention shifts in response to discriminative cues. To emulate the iterative observation process, we develop a Context-guided Iterative Optimization Framework (CIOF) that enables each iteration’s predictions to inform subsequent feature processing, creating a feedback loop that steadily refines detection results through global context integration and targeted feature enhancement.

Our main contributions can be summarized as follows:

- We propose IRFNet, a novel iterative refinement framework for camouflaged object detection that mimics human visual cognition through progressive feature enhancement and feedback mechanisms.
- We design a hierarchical feature enhancement module that effectively integrates multiscale features through bilateral and trilateral fusion pathways. This is coupled with a dynamic channel-spatial attention mechanism for adaptive feature modulation.
- We develop a context-guided iterative optimization framework that combines global semantic guidance with iterative refinement, enabling comprehensive scene understanding and progressive prediction improvement through feedback integration and dual branch supervision.
- We conduct comprehensive comparisons with state-of-the-art methods and demonstrate consistent superiority across evaluation metrics on three challenging benchmark datasets. Additionally, our approach shows strong generalization capability when applied to medical polyp segmentation, confirming its practical value across different domains.

## 2. Related Work

### 2.1. Camouflaged Object Detection

Camouflaged object detection has evolved significantly, from early methods using hand-crafted features to modern deep learning approaches. Early works relied on low-level visual cues such as color [15], texture [16], and intensity [17], but often struggled with complex scenes. The introduction of large-scale datasets such as CAMO [18], COD10K [19], and NC4K [20] catalyzed the development of deep learning-based methods, significantly improving detection accuracy.

Recent deep learning approaches can be broadly categorized based on their key strategies. Multiscale feature integration approaches such as ZoomNet [21] and C2FNet [22] address the challenge of detecting camouflaged objects at varying scales by fusing features across different levels of feature hierarchies. Boundary-aware methods such as BGNet [23] and FEDER [24] incorporate explicit edge information to improve segmentation accuracy at object boundaries. Several methods leverage uncertainty modeling [25,26] to handle ambiguous regions, while others adopt graph-based reasoning [27] to capture complex relationships between image regions. Despite these advances, fully addressing the unique challenges of camouflaged object detection remains an open problem.

### 2.2. Feature Fusion and Attention Mechanisms

Feature fusion architectures have been widely adopted in medical image segmentation and camouflaged object detection. U-Net-based architectures [28,29] employ skip connections to fuse low-level and high-level features. PraNet [11] introduces a parallel reverse attention mechanism using features from the last three stages while neglecting finer details from higher-resolution features. C2FNet [22] employs context-aware cross-level fusion with a combination of maximum pooling and point-wise convolutions, although its inter-layer fusion remains relatively simple and focuses primarily on high-level features.

In contrast to these approaches, our Hierarchical Feature Enhancement Module (HFEM) first fuses features through bilateral and trilateral pathways that establish connections between adjacent layers, then enhances these fused features using our proposed attention mechanism. Unlike existing attention mechanisms, our Dynamic Channel-Spatial Attention (DCSA) module incorporates multiple pooling operations (average, maximum, and mixed) with dynamic weighting in its channel attention component, significantly enhancing attention representation compared to single maximum pooling approaches. We further differentiate our approach through a spatial attention mechanism incorporating multiscale dilated convolutions that capture features at different receptive fields.

### 2.3. Iterative Refinement Strategies

Iterative refinement has shown promising results in various vision tasks by allowing progressive improvement through multiple processing passes. TPRNet [30] implements a progressive refinement approach based on the Res2Net [31] architecture, but lacks an explicit feedback mechanism, limiting its ability to learn from previous predictions. HitNet [32], built on PVT-v2 [33], introduces an iterative feedback mechanism, but primarily applies it to low-resolution features while maintaining separate processing streams for high and low-resolution features. This separation potentially limits the benefits of iterative refinement for fine-grained segmentation.

Departing from these designs, our approach implements a unified architecture across all feature levels and strategically places feedback at the highest-resolution layer, where fine-grained features are most beneficial for precise segmentation.

## 3. Materials and Methods

### 3.1. Network Architecture Overview

**Figure 1:**
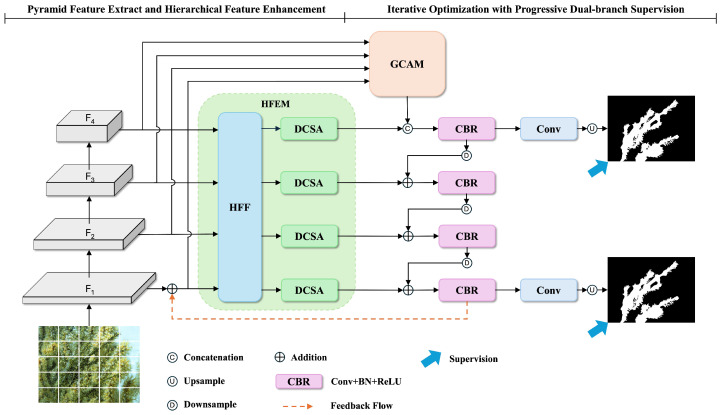
Overview of our proposed network architecture.

As shown in Figure 1, given an input image $I\in R^{H\times W\times 3}$, we first employ PVT-v2-B2 [33] as the backbone to extract multiscale features ${\left\{F_{i}\right\}}_{i=1}^{4}$ with resolutions of $\frac{H}{2^{i+1}}\times \frac{W}{2^{i+1}}$. These features are then processed through a channel reduction module to obtain unified feature representations ${\left\{X_{i}\right\}}_{i=1}^{4}$ with channel dimension C=64. To effectively leverage these multiscale features, we design two collaborative modules that work in an iterative manner: (1) a Hierarchical Feature Enhancement Module (HFEM) that enriches feature representations through multiscale fusion and dynamic attention at each iteration stage; (2) a Context-guided Iterative Optimization Framework (CIOF) that integrates global context through the Global Context Awareness Module (GCAM) before iteratively refining features through parallel prediction pathways, with one path focused on global semantic understanding and the other on refined local details. In addition, a feedback mechanism is incorporated to utilize previous iteration results for continuous improvement.

### 3.2. Hierarchical Feature Enhancement Module

#### 3.2.1. Hierarchical Feature Fusion

**Figure 2:**
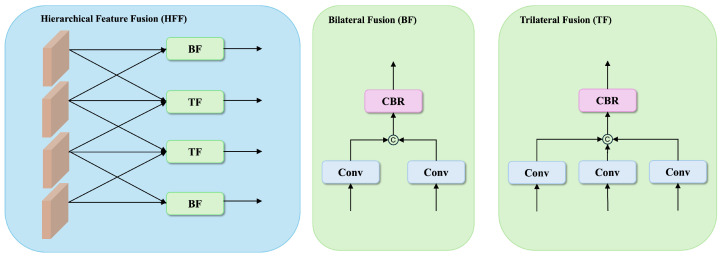
Illustration of our proposed Hierarchical Feature Fusion (HFF) module.

Given backbone features ${\left\{X_{i}\right\}}_{i=1}^{4}$ after channel reduction, we design a hierarchical feature fusion mechanism that combines both bilateral and trilateral fusion pathways to comprehensively capture cross-scale relationships, as illustrated in Figure 2.

For bilateral fusion, we establish connections between feature pairs

(1) $$F_{i,j}=\mathrm{CBR}\left(\left[{\mathrm{CONV}}_{3\times 3}\left(X_{i}\right),{\mathrm{CONV}}_{3\times 3}\left(X_{j}\right)\right]\right)$$

where [·,·] denotes channel-wise concatenation, CBR represents a sequential combination of convolution, batch normalization, and ReLU activation, and both feature maps are aligned to the same spatial dimensions through bilinear interpolation when necessary. Specifically, we compute bilateral fusion features between two adjacent levels, resulting in $F_{1,2}$ and $F_{3,4}$.

For trilateral fusion, we integrate features from three consecutive scales:

(2) $$F_{i,j,k}=\mathrm{CBR}\left(\left[{\mathrm{CONV}}_{3\times 3}\left(X_{i}\right),{\mathrm{CONV}}_{3\times 3}\left(X_{j}\right),{\mathrm{CONV}}_{3\times 3}\left(X_{k}\right)\right]\right)$$

This results in two trilateral fusion features $F_{1,2,3}$ and $F_{2,3,4}$, capturing multiscale contextual information at different semantic levels.

#### 3.2.2. Dynamic Channel-Spatial Attention

To effectively capture both channelwise and spatial feature dependencies while maintaining adaptability to different scales of information, we propose the Dynamic Channel-Spatial Attention (DCSA) module to enhance the hierarchically fused features from HFF.

**Figure 3:**
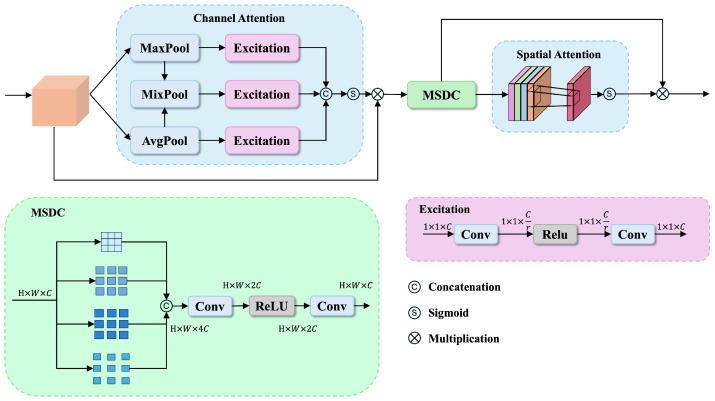
Illustration of our proposed Dynamic Channel-Spatial Attention (DCSA) module.

As illustrated in Figure 3, the DCSA consists of three key components: (1) Channel attention with dynamic pooling that adaptively integrates global average and maximum information, followed by Excitation modules consisting of two 1×1 convolution layers with a ReLU activation in between to capture channel dependencies; (2) Multi-scale dilated convolution (MSDC) that captures features at different scales through parallel dilated convolutions; (3) Spatial attention that emphasizes informative spatial regions. These components work together synergistically to enhance feature representations.

Given an input feature map $F_{i,j}$ or $F_{i,j,k}$ from HFF module $X\in R^{C\times H\times W}$, we first apply three parallel pooling operations to capture different aspects of channelwise statistics:

(3) $$\begin{array}{cc} z_{\mathrm{avg}} & =\mathrm{AdaptiveAvgPool}2d(X)\\ z_{\max} & =\mathrm{AdaptiveMaxPool}2d(X)\\ z_{\mathrm{mix}} & =\alpha z_{\mathrm{avg}}+\left(1-\alpha \right)z_{\max} \end{array}$$

where α=0.5 balances the contributions of different pooling strategies.

These pooled features are then processed through a shared two-layer network implemented with 1 × 1 convolutions

(4) $${\mathrm{CONV}}_{\mathrm{exc}}\left(z\right)=\mathrm{sigmoid}\left({\mathrm{CONV}}_{1\times 1}\left(\mathrm{ReLU}\left({\mathrm{CONV}}_{1\times 1}\left(z\right)\right)\right)\right),$$

where the first ${\mathrm{CONV}}_{1\times 1}$ reduces the channel dimension from *C* to C/r (with *r* as the reduction ratio) and the second ${\mathrm{CONV}}_{1\times 1}$ restores it back to *C*. The final channel attention map is computed as follows:

(5) $$M_{c}=\mathrm{sigmoid}\left({\mathrm{CONV}}_{\mathrm{exc}}\left(z_{\mathrm{avg}}\right)+{\mathrm{CONV}}_{\mathrm{exc}}\left(z_{\max}\right)+{\mathrm{CONV}}_{\mathrm{exc}}\left(z_{\mathrm{mix}}\right)\right).$$

Following the channel attention, we employ a Multi-Scale Dilated Convolution (MSDC) module to capture multiscale contextual information. The MSDC module applies four parallel dilated convolutions with different dilation rates

(6) $$D_{i}=\mathrm{ReLU}\left(\mathrm{BN}\left({\mathrm{DConv}}_{3\times 3}\left(X⊙M_{c},r_{i}\right)\right)\right),r_{i}\in \left\{1,3,5,7\right\},$$

where ${\mathrm{DConv}}_{3\times 3}\left(\cdot ,r_{i}\right)$ represents dilated convolution with kernel size 3 × 3 and dilation rate $r_{i}$. The multiscale features are then concatenated and processed through the following dimension reduction operation:

(7) $$F_{m}=\mathrm{ReLU}\left(\mathrm{BN}\left({\mathrm{CONV}}_{1\times 1}\left(\left[D_{1},D_{3},D_{5},D_{7}\right]\right)\right)\right).$$

For spatial attention, we exploit interchannel relationships by first computing the channel-wise statistics:

(8) $$\begin{array}{cc} s_{\mathrm{avg}} & =\frac{1}{C}\sum_{i=1}^{C}F_{m}^{i}\\ s_{\max} & =\max_{i=1}^{C}F_{m}^{i} \end{array}$$

where $F_{m}^{i}$ represents the *i*-th channel of $F_{m}$. The spatial attention map is then generated through

(9) $$M_{s}=\mathrm{sigmoid}\left({\mathrm{CONV}}_{7\times 7}\left(\left[s_{\mathrm{avg}},s_{\max}\right]\right)\right).$$

The final output of the DCSA module is computed as

(10) $$Y=F_{m}⊙M_{s}.$$

The features enhanced by DCSA, that is, the output of the HFEM module, are then used for the top-down refinement path.

### 3.3. Context-Guided Iterative Optimization Framework

#### 3.3.1. Global Context Awareness Module

**Figure 4:**
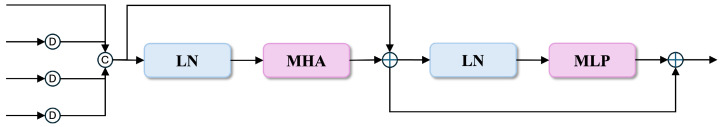
Architecture of our Global Context Awareness Module (GCAM).

As shown in Figure 4, given backbone features ${\left\{F_{i}\right\}}_{i=1}^{4}$, we first downsample and concatenate them to obtain a unified feature representation

(11) $$X=\mathrm{Concat}[\mathrm{Down}\left(F_{1}\right),\mathrm{Down}\left(F_{2}\right),\mathrm{Down}\left(F_{3}\right),F_{4}],$$

where Down represents downsampling operations that align features to the spatial dimensions of $F_{4}$. The concatenated feature $X\in R^{C\times H\times W}$ is then reshaped to a sequence of tokens $X_{s}\in R^{HW\times C}$ for attention computation.

The GCAM processes these tokens through two main blocks. The first block applies multi-head attention as follows:

(12) $$Y_{1}=\mathrm{MHA}\left(\mathrm{LN}\left(X_{s}\right)\right)+X_{s}$$

where LN denotes layer normalization and MHA represents multi-head attention.

The second block further processes the attention output through a multilayer perceptron:

(13) $$Y_{2}=\mathrm{MLP}\left(\mathrm{LN}\left(Y_{1}\right)\right)+Y_{1}$$

where the MLP consists of two linear transformations with a GELU activation in between:

(14) $$\mathrm{MLP}\left(X\right)={\mathrm{Linear}}_{2}\left(\mathrm{GELU}\left({\mathrm{Linear}}_{1}\left(X\right)\right)\right).$$

Finally, the processed features are reshaped back to the spatial domain to obtain the global context representation $g\in R^{C\times H\times W}$.

#### 3.3.2. Iterative Optimization Mechanism

We design an iterative optimization mechanism that combines global semantic guidance with local detail enhancement. At each iteration *t*, our mechanism first enhances the initial feature representation by incorporating feedback from the previous iteration:

(15) $$f_{1}^{t}=\left\{\begin{array}{cc} F_{1}, & t=1\\ F_{1}+\phi \left(r_{1}^{t-1}\right), & t>1 \end{array}\right.$$

where $F_{1}$ is the high-resolution feature from the backbone, $r_{1}^{t-1}$ represents the refined feature from the previous iteration, and ϕ(·) denotes the upsampling operation.

The enhanced feature $f_{1}^{t}$ together with other backbone features ${\left\{F_{i}\right\}}_{i=2}^{4}$ are processed by our previously described Hierarchical Feature Enhancement Module (HFEM) to obtain the enhanced multiscale representations ${\left\{e_{i}^{t}\right\}}_{i=1}^{4}$.

We employ the GCAM to capture the global context, which is then utilized to enhance the highest-level features before starting the top-down refinement process:

(16) $$g^{t}=\mathrm{GCAM}\left(\left[f_{1}^{t},F_{2},F_{3},F_{4}\right]\right),$$

(17) $$r_{4}^{t}=\mathrm{CBR}\left(\mathrm{Concat}\left[e_{4}^{t},g^{t}\right]\right).$$

The refinement pathway then progressively propagates semantic information while preserving fine details:

(18) $$r_{i-1}^{t}=\mathrm{CBR}\left(e_{i}^{t}+\mathrm{Down}\left(r_{i}^{t}\right)\right),\;i\in \left\{4,3,2\right\},$$

Finally, we generate predictions through two complementary branches:

(19) $$P_{\mathrm{global}}^{t}=\mathrm{Conv}\left(r_{4}^{t}\right),$$

(20) $$P_{\mathrm{refine}}^{t}=\mathrm{Conv}\left(r_{1}^{t}\right).$$

Both prediction maps are upsampled to the input resolution for supervision.

#### 3.3.3. Loss Function

To effectively supervise our network, we employ a structure-aware loss function combined with a progressive supervision strategy. For each prediction $P^{t}$ and ground truth mask G, we compute

(21) $$L_{\mathrm{struc}}\left(P^{t},G\right)=L_{\mathrm{wBCE}}\left(P^{t},G\right)+L_{\mathrm{wIoU}}\left(P^{t},G\right),$$

where $L_{\mathrm{wBCE}}$ denotes the weighted binary cross-entropy loss that emphasizes boundary regions and $L_{\mathrm{wIoU}}$ measures the weighted intersection over union between the prediction and ground truth.

To encourage progressive refinement through iterations, we define the total loss as

(22) $$L_{\mathrm{total}}=\sum_{t=1}^{T}\gamma t\left(L_{\mathrm{struc}}\left(P_{\mathrm{global}}^{t},G\right)+L_{\mathrm{struc}}\left(P_{\mathrm{refine}}^{t},G\right)\right),$$

where γ=0.05 controls the weights of different iterations, encouraging the network to continuously refine its predictions.

## 4. Experiments

### 4.1. Implementation Details

We implemented our model using PyTorch 2.5.1 and conducted all experiments on a single NVIDIA A40 GPU (48GB) with CUDA 12.4. Additional computational efficiency tests were performed on an NVIDIA RTX 4090 consumer-grade GPU. Our experiments were performed on a system with an AMD EPYC 7543 32-core processor running Ubuntu 22.04. For the backbone network, we employed PVT-v2-B2 [33] pretrained on ImageNet. During training, we employed an effective batch size of 16 and trained the model for 100 epochs. We used the AdamW optimizer with an initial learning rate of $1\times 10^{-4}$ and weight decay of $1\times 10^{-4}$. The learning rate was adjusted using a step scheduler that decayed by a factor of 0.1 every 30 epochs. To prevent gradient explosion, we applied gradient clipping with a maximum norm of 0.5. For data preprocessing, input images were resized to a resolution of 704 × 704 pixels. During training, we employed standard data augmentation techniques to enhance model generalization, including random horizontal flipping, random rotation, and color jittering. No augmentation was applied during the testing phase. The complete training process took approximately 12 h, with each epoch requiring around 7 min.

### 4.2. Datasets

We evaluated our proposed IRFNet on three widely-used camouflaged object detection benchmark datasets:

CAMO [18] contains 1250 images across eight categories, including both natural and artificial camouflage examples. The dataset is split into 1000 training images and 250 testing images.

COD10K [19] features 5066 high-quality images across 78 categories, covering aquatic, terrestrial, flying animals, and artificial camouflage art, which are divided into 3040 training images and 2026 testing images with detailed annotations.

NC4K [20] comprises 4121 high-quality images with instance-level annotations, serving as the largest COD test dataset to evaluate model generalization.

Following established protocol [19], we used a combined training set of 4040 images (1000 from CAMO and 3040 from COD10K) and performed evaluation on all three test sets.

### 4.3. Evaluation Metrics

To comprehensively evaluate our model’s performance, we adopted four widely used metrics in camouflaged object detection: the structure measure ($S_{\alpha }$) [34], mean absolute error (MAE) [35], enhanced-alignment measure ($E_{\phi }$) [36], and weighted F-measure ($F_{\beta }^{w}$) [37].

The structure measure $S_{\alpha }$ evaluates the structural similarity between predictions and ground truth considering both region-aware and object-aware structural similarities. The MAE computes the average absolute pixel-wise difference between the predicted mask and ground truth, with lower values indicating better performance. The $E_{\phi }$ considers both local and global similarities; we report the mean E-measure ($E_{\phi }^{mean}$). The $F_{\beta }^{w}$ combines precision and recall with $\beta^{2}=0.3$, which emphasizes precision over recall.

For all metrics except MAE, higher values indicate better performance. We follow standard evaluation protocols established in previous works in order to ensure fair comparison.

### 4.4. Comparison with State-of-the-Art Methods

To demonstrate the effectiveness of our proposed IRFNet, we conducted comprehensive comparisons with fourteen state-of-the-art (SOTA) COD models, including SINet [19], TINet [38], C2FNet [22], PFNet [39], SegMaR [40], BGNet [23], PreyNet [41], SINetV2 [42], ZoomNet [21], HitNet [32], FEDER [24], CFANet [43], PopNet [44], and VSCode [45]. For fair comparison, all models were evaluated on the same benchmark datasets using identical evaluation metrics.

**Figure 5:**
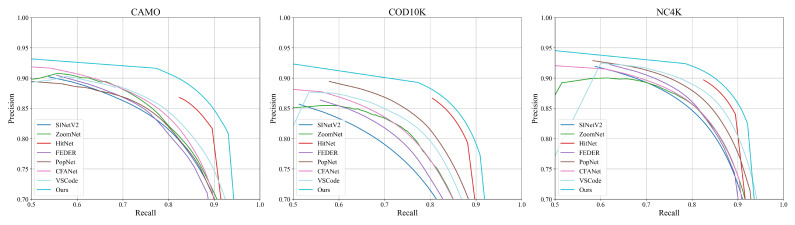
Comparison of precision–recall curves on three benchmark datasets.

**Quantitative Results.** As shown in Table 1, our proposed IRFNet consistently outperforms existing methods across all datasets. Compared to HitNet [32], IRFNet achieves average improvements of 13.7% in MAE, 1.8% in $S_{\alpha }$, 0.9% in $E_{\phi }$, and 1.8% in $F_{\beta }^{w}$ across all three datasets. When compared to the recent VSCode [45], our method demonstrates even more significant improvements, with average gains of 22.0% in MAE, 3.3% in $S_{\alpha }$, 2.5% in $E_{\phi }$, and 7.3% in $F_{\beta }^{w}$. The precision–recall curves in Figure 5 further illustrate our method’s superior detection performance, consistently maintaining higher precision across varying recall values. These substantial improvements across different metrics and datasets validate the effectiveness of our proposed approach.

**Figure 6:**
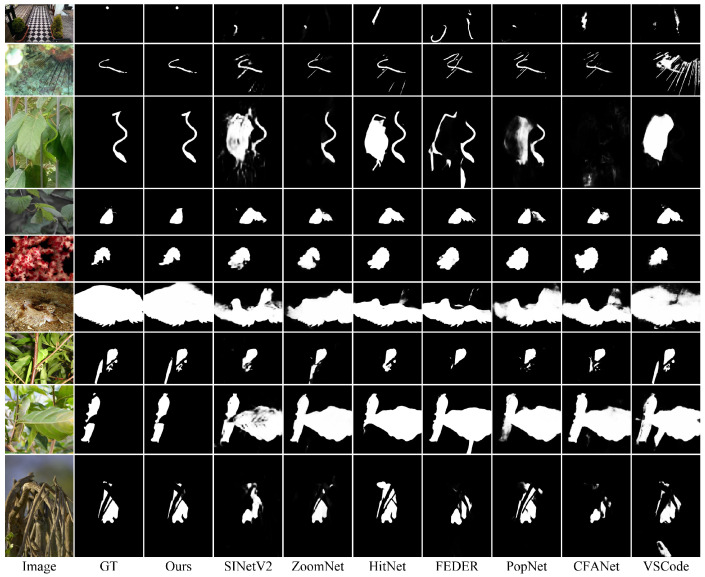
Visual comparison with state-of-the-art methods. Our proposed IRFNet generates more accurate and complete segmentation maps across various challenging scenarios.

**Qualitative Results.** The visual comparisons in Figure 6 demonstrate IRFNet’s superior performance across challenging scenarios. For extremely fine-grained objects with minimal spatial extent (rows 1–3), our method successfully identifies targets that competing approaches struggle with. In cases of elongated structures such as snakes (rows 2–3), IRFNet accurately captures subtle occlusions and fine tail details that methods like ZoomNet [21] fail to detect. For medium-sized targets (rows 4–5), where other approaches mistakenly incorporate background leaves as part of the insect objects, our network maintains precise object boundaries. For larger objects such as the fish in row 6, it can be seen that existing methods only detect partial body segments, while our approach captures the complete structure. The bottom rows (7–9) showcase IRFNet’s capability in handling complex occlusion scenarios, where existing methods either misclassify occluding elements as part of the target or segment only portions of the object due to intermediate occlusions.

**Computational Efficiency.** We evaluated IRFNet’s computational efficiency against recent state-of-the-art methods, with the results shown in Table 2. All measurements were conducted on an NVIDIA RTX 4090 GPU. Compared to recent methods such as HitNet [32], FEDER [24], and PopNet [44], our approach maintains a balanced profile of parameters, computational complexity, inference speed, and memory consumption. While our iterative refinement introduces some computational overhead compared to single-pass methods such as CFANet [43], IRFNet still achieves competitive efficiency metrics while delivering superior detection accuracy. This balance between efficiency and performance makes IRFNet particularly suitable for applications where detection quality is prioritized, such as medical analysis and precision inspection systems.

### 4.5. Ablation Study

**Figure 7:**
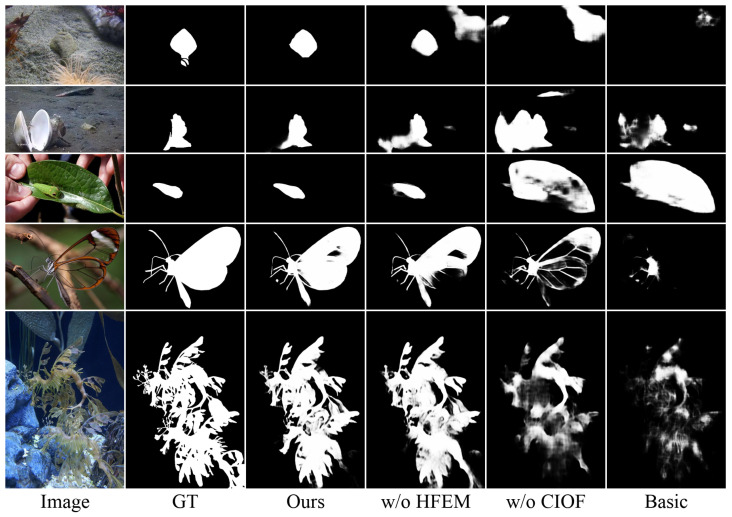
Qualitative comparison of different model variants.

**Effectiveness of Proposed Modules.** To comprehensively evaluate the contributions of each module, we conducted ablation studies by examining three variants: removing the HFEM, removing CIOF, and removing both modules to form the basic model. As shown in Table 3, removing CIOF causes significant performance degradation, with average increases of 27.3% in MAE, 2.5% in $S_{\alpha }$, 2.3% in $E_{\phi }$, and 4.6% in $F_{\beta }^{w}$ across all datasets. In contrast, removing the HFEM leads to relatively milder degradation, with average increases of 12.1% in MAE, 0.8% in $S_{\alpha }$, 0.5% in $E_{\phi }$, and 1.3% in $F_{\beta }^{w}$. When both modules are removed the performance drops substantially, with average degradation of 36.0% in MAE, 6.5% in $S_{\alpha }$, 7.2% in $E_{\phi }$, and 11.4% in $F_{\beta }^{w}$. The qualitative comparisons in Figure 7 further validate these findings. Without the HFEM, the predictions exhibit a slight degradation in detail preservation, while removing CIOF leads to more severe performance drops in the form of blurred prediction maps due to failed object localization and increased prediction uncertainty. Notably, while both components contribute positively to the overall performance, the more significant performance degradation observed when removing the CIOF module indicates its particularly crucial role in accurate camouflaged object detection.

**Figure 8:**
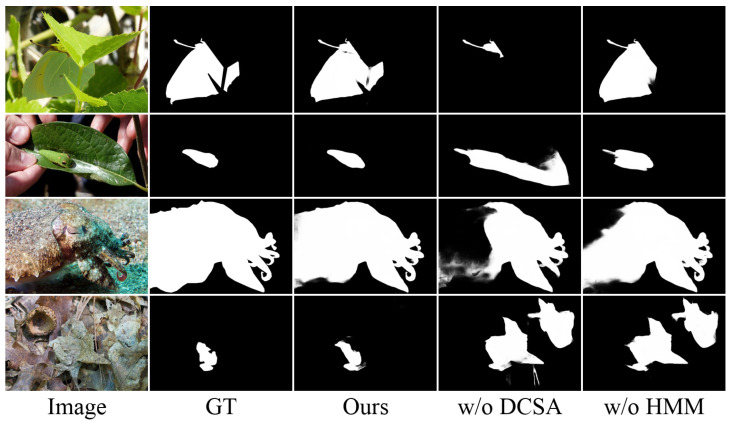
Qualitative comparison of HFEM variants.

**Effectiveness of HFEM Components.** To evaluate the contribution of the individual components within the HFEM, we conducted ablation experiments on the Hierarchical Multiscale Module (HMM) and Dynamic Channel-Spatial Attention (DCSA). As shown in Table 4, removing the DCSA module leads to average performance degradation of 7.7% in MAE, 0.5% in $S_{\alpha }$, 0.3% in $E_{\phi }$, and 0.7% in $F_{\beta }^{w}$ across all datasets. Similarly, removing the HMM causes average degradation of 6.2% in MAE, 0.4% in $S_{\alpha }$, 0.4% in $E_{\phi }$, and 0.7% in $F_{\beta }^{w}$. The visual comparisons in Figure 8 reveal distinct failure patterns; removing DCSA leads to more diffused predictions with uncertain boundaries, while absence of the HMM tends to cause over-smoothed results. These patterns align with our design intuition, as DCSA dynamically modulates features, while the HMM enables multiscale integration. Notably, while removing individual components can cause specific types of detection errors, their combination works synergistically to produce surprisingly robust predictions, demonstrating the effectiveness of their coordinated feature enhancement.

**Figure 9:**
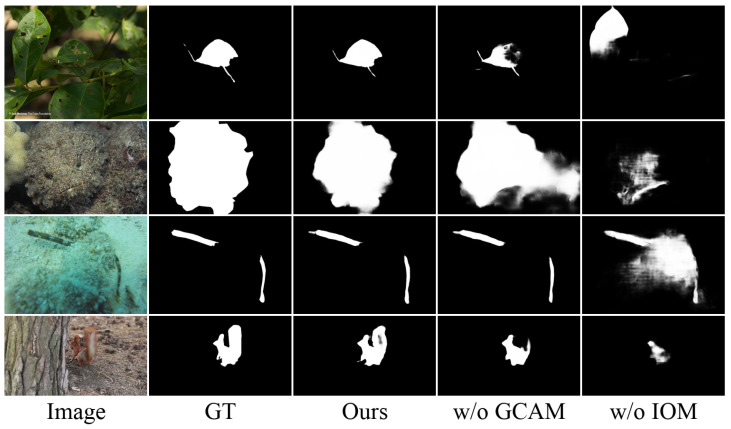
Qualitative comparison of CIOF components.

**Effectiveness of CIOF Components.** The results of our ablation studies reveal the distinct contributions of the GCAM and IOM within CIOF. As shown in Table 5, the IOM proves to be the more critical component, with its removal causing average performance drops of 25.2% in MAE, 2.2% in $S_{\alpha }$, 1.9% in $E_{\phi }$, and 4.3% in $F_{\beta }^{w}$ across all datasets. This degradation is particularly pronounced on the CAMO dataset, where the MAE increases from 0.047 to 0.059 and $F_{\beta }^{w}$ drops from 0.830 to 0.796. The GCAM’s contribution, while still significant, shows more moderate impact; its removal leads to average degradation of 5.6% in MAE, 0.4% in $S_{\alpha }$, 0.2% in $E_{\phi }$, and 0.6% in $F_{\beta }^{w}$. These quantitative findings align with our qualitative observations in Figure 9 in that absence of the GCAM manifests primarily in incomplete object detection, as the global context is crucial in this regard, while removing the IOM leads to more fundamental issues including incorrect object localization, imprecise boundaries, and poor object-background separation.

**Effect of Different Backbone Networks.** To investigate the impact of different backbone architectures, we evaluated our framework using various networks: ResNet-50 [46], Res2Net-50 [31], ConvNeXt-Base [47], Swin Transformer-Small [48], and PVT-v2-B2 [33]. As shown in Table 6, modern transformer-based architectures consistently outperform traditional CNN backbones across all datasets. While ConvNeXt-Base, Swin-S, and PVT-v2-B2 achieve comparable detection accuracy, PVT-v2-B2 offers a significantly better efficiency–performance tradeoff, requiring fewer FLOPs and maintaining a moderate parameter count. This favorable balance influenced our selection of PVT-v2-B2 as the default backbone.

**Ablation of Iteration Numbers.** As shown in Table 7, we conducted experiments with varying iteration counts (1–5). Our results clearly demonstrate that three iterations achieves optimal performance compared to other total iteration numbers. For example, on the CAMO dataset, the MAE decreases from 0.054 to 0.047 when increasing from one to three iterations, then increases to 0.054 and 0.056 with four and five iterations, respectively. This pattern appears consistently across all datasets, indicating that excessive iterations may lead to overfitting or error accumulation rather than continued improvement.

**Figure 10:**
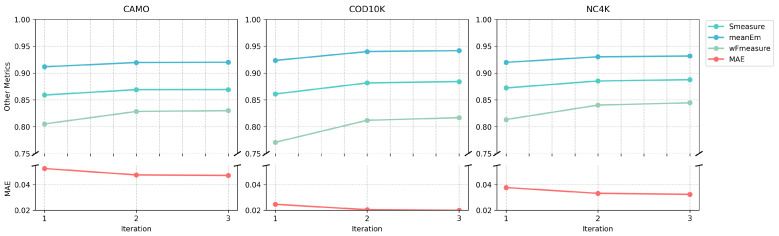
Evolution of performance metrics during the iterations in our optimal three-iteration model on the CAMO, COD10K, and NC4K datasets. The plots demonstrate consistent improvement across iterations, with MAE decreasing and other metrics increasing.

**Figure 11:**
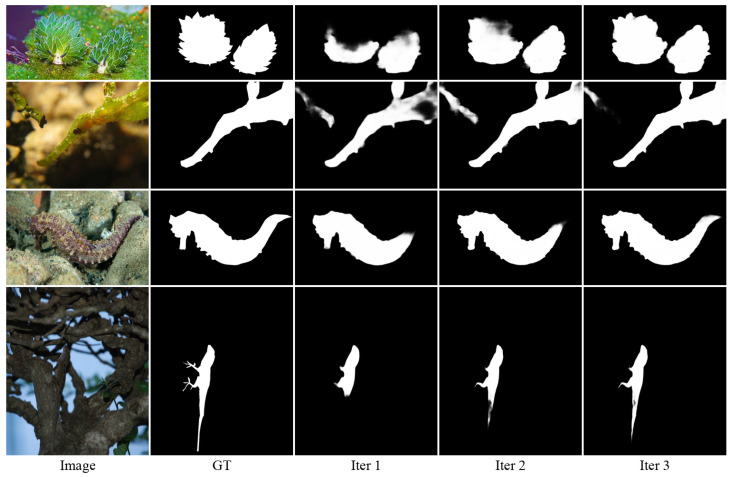
Visualization of the iterative refinement process in our optimal three-iteration model. From left to right: input image, ground truth (GT), and prediction after first iteration (Iter 1), second iteration (Iter 2), and third iteration (Iter 3). The prediction quality progressively improves with each iteration.

Given this finding, we further analyzed the progressive improvement within the three iterations of our optimal model. Table 8 shows the detailed progression of metrics through iterations, revealing consistent improvements across all datasets. For example, the MAE decreases from 0.053 to 0.047 on CAMO, 0.025 to 0.020 on COD10K, and 0.038 to 0.032 on NC4K. Figure 10 visualizes these improvements for all evaluation metrics, while Figure 11 provides qualitative examples showing how the prediction quality improves during iterations.

**Effectiveness of Dual-Branch Supervision Strategy.** We compared three approaches for supervision: using only the refined branch prediction, combining both branch predictions before supervision, and using our proposed approach of supervising both branches separately. As shown in Table 9, single-branch supervision achieves reasonable but limited results, with an MAE of 0.055 on CAMO and 0.021 on COD10K. While the combined branch approach shows improvements in $S_{\alpha }$, our dual-branch strategy achieves the best overall performance, with lower MAE, higher $E_{\phi }$, and higher $F_{\beta }^{w}$ across all datasets. These results demonstrate that separately supervising the global and refined predictions enables both more effective learning of semantic understanding and finer detail preservation.

### 4.6. Application to Polyp Segmentation

To demonstrate the generalizability of our proposed IRFNet beyond natural camouflage scenarios, we evaluated its performance on a medical image segmentation task, specifically, polyp segmentation. Polyps are a precursor to colorectal cancer, and exhibit high visual similarity to the surrounding normal colonic mucosa. Their early detection through accurate segmentation in colonoscopy images is crucial for preventive care.

#### 4.6.1. Experimental Settings

Following established protocols, we trained IRFNet using 1450 images from the Kvasir [49] and CVC-ClinicDB [50] datasets. We then evaluated its performance on five test sets consisting of the remaining 100 images from Kvasir, and four independent datasets: CVC-300 [51] (60 images), CVC-ClinicDB (553 images), CVC-ColonDB [52] (380 images), and ETIS-LaribPolypDB [53] (196 images). During training, images were resized to 352 × 352 pixels with standard augmentation techniques (random rotation and flipping). The model was trained for 60 epochs with a learning rate of $1\times 10^{-4}$ and weight decay of $1\times 10^{-4}$ using the AdamW optimizer, which it completed in approximately one hour.

#### 4.6.2. Comparison with State-of-the-Art Methods

Table 10 presents a comprehensive comparison between our proposed IRFNet and five state-of-the-art polyp segmentation methods: UNet [28], UNet++ [29], SFA [10], PraNet [11], and CFANet [12]. Our approach demonstrates superior performance across all datasets and metrics, with particularly significant improvements on challenging cases in ETIS-LaribPolypDB and CVC-ColonDB. Specifically, compared to the second-best performer (CFANet), IRFNet achieves improvements of 3.5% in $S_{\alpha }$, 2.4% in $E_{\phi }$, and 8.8% in $F_{\beta }^{w}$ on ETIS-LaribPolypDB and 4.5% in $S_{\alpha }$, 5.3% in $E_{\phi }$, and 9.5% in $F_{\beta }^{w}$ on CVC-ColonDB.

**Figure 12:**
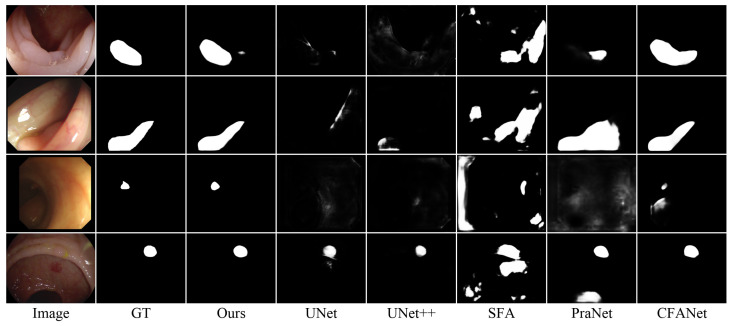
Visual comparison of polyp segmentation results.

Figure 12 presents visual comparisons across various challenging polyp segmentation scenarios. IRFNet demonstrates superior performance in three critical aspects: (1) accurately delineating boundaries between polyps and surrounding tissue, (2) effectively detecting small polyps that are missed or oversegmented by other methods, and (3) maintaining robust performance under varying lighting conditions. The consistent improvements across different polyp sizes and appearances demonstrate that the hierarchical feature enhancement and iterative refinement mechanisms in IRFNet effectively generalize to medical imaging contexts where target–background similarity poses significant challenges.

These results validate the conclusion that our proposed cognitive-inspired iterative refinement approach effectively addresses the fundamental challenge of distinguishing visually similar structures in natural camouflage as well as in medical imaging contexts, confirming the broader applicability of our method beyond its original design domain.

## 5. Conclusions

This paper proposes IRFNet, a novel framework for camouflaged object detection that effectively addresses the challenges of detecting objects intentionally hidden within their surroundings. Our framework’s key components consist of a Hierarchical Feature Enhancement Module (HFEM) and a Context-guided Iterative Optimization Framework (CIOF), which work together synergistically to enrich multiscale feature representations and progressively refine predictions through global context and local detail integration. Through extensive experiments on three challenging benchmark datasets (CAMO, COD10K, and NC4K), our results demonstrate that IRFNet consistently outperforms state-of-the-art methods, demonstrating high effectiveness.

While achieving superior detection accuracy, our iterative refinement mechanism requires additional computational resources compared to single-forward-pass methods. Nevertheless, IRFNet maintains competitive efficiency on modern hardware. This balance between efficiency and accuracy could be further improved through future research on lightweight attention mechanisms and model compression techniques. Moreover, as GPU hardware continues to advance, the computational demands of iterative approaches will become increasingly manageable.

Looking forward, promising research directions include developing adaptive iteration mechanisms that dynamically determine refinement steps based on image complexity, similar to how large language models adjust reasoning depth according to task difficulty. Another direction involves exploring backtracking mechanisms inspired by cognitive processes that can re-evaluate decisions made in previous iterations. Additionally, extending our approach to emerging tasks such as Referring Camouflaged Object Detection (Ref-COD) [54] represents a valuable future direction. Such an approach would enable precise identification of specific camouflaged targets through reference image matching, which is particularly valuable in ecological monitoring, medical diagnosis, and other domains requiring targeted detection in complex environments. Such targeted approaches may also reduce dependency on extensive general training datasets while maintaining robust detection performance.

## Figures and Tables

**Table 1:** Quantitative comparison with state-of-the-art methods. The best and second-best results are highlighted in **bold** and underline, respectively; ↑ indicates higher is better; ↓ indicates lower is better.

Method	Publication	CAMO				COD10K				NC4K			
		M↓	$S_{\alpha }↑$	$E_{\phi }↑$	$F_{\beta }^{w}↑$	M↓	$S_{\alpha }↑$	$E_{\phi }↑$	$F_{\beta }^{w}↑$	M↓	$S_{\alpha }↑$	$E_{\phi }↑$	$F_{\beta }^{w}↑$
SINet [19]	CVPR’20	0.092	0.745	0.804	0.644	0.043	0.776	0.864	0.631	0.058	0.808	0.871	0.723
TINet [38]	AAAI’21	0.087	0.781	0.836	0.678	0.042	0.793	0.861	0.635	0.055	0.829	0.879	0.734
C2FNet [22]	IJCAI’21	0.080	0.796	0.854	0.719	0.036	0.813	0.890	0.686	0.049	0.838	0.897	0.762
PFNet [39]	CVPR’21	0.085	0.782	0.841	0.695	0.040	0.800	0.877	0.660	0.053	0.829	0.887	0.745
SegMaR [40]	CVPR’22	0.071	0.815	0.874	0.753	0.034	0.833	0.899	0.724	0.046	0.841	0.896	0.781
BGNet [23]	IJCAI’22	0.073	0.812	0.870	0.749	0.033	0.831	0.901	0.722	0.044	0.851	0.907	0.788
PreyNet [41]	MM’22	0.077	0.790	0.842	0.708	0.034	0.813	0.881	0.697	0.050	0.834	0.887	0.763
SINetV2 [42]	TPAMI’22	0.070	0.820	0.882	0.743	0.037	0.815	0.887	0.680	0.048	0.847	0.903	0.770
ZoomNet [21]	CVPR’22	0.066	0.820	0.877	0.752	0.029	0.838	0.888	0.729	0.043	0.853	0.896	0.784
HitNet [32]	AAAI’23	0.055	0.849	0.906	0.809	0.023	0.871	0.935	0.806	0.037	0.875	0.926	0.834
FEDER [24]	CVPR’23	0.071	0.802	0.867	0.738	0.032	0.822	0.900	0.716	0.044	0.847	0.907	0.789
CFANet [43]	ICME’23	0.073	0.815	0.869	0.751	0.031	0.834	0.897	0.728	0.046	0.848	0.897	0.782
PopNet [44]	ICCV’23	0.077	0.808	0.859	0.744	0.028	0.851	0.910	0.757	0.042	0.861	0.909	0.802
VSCode [45]	CVPR’24	0.060	0.836	0.892	0.768	0.028	0.847	0.913	0.744	0.038	0.874	0.920	0.813
IRFNet (Ours)		**0.047**	**0.869**	**0.920**	**0.830**	**0.020**	**0.884**	**0.942**	**0.817**	**0.032**	**0.888**	**0.932**	**0.845**

**Table 2:** Comparison of computational efficiency with state-of-the-art methods.

Method	PFNet [39]	BGNet [23]	PreyNet [41]	ZoomNet [21]	HitNet [32]	FEDER [24]	CFANet [43]	PopNet [44]	Ours
FLOPs (G)	53.2	116.9	116.2	203.8	140.3	68.6	52.5	455.8	203.4
Params (M)	46.5	79.9	38.5	32.4	26.2	44.1	29.2	223.9	27.2
FPS	76.6	81.2	71.5	38.2	52.1	22.6	64.9	18.7	39.6
Memory (MB)	377.9	507.1	473.1	496.7	318.8	367.2	290.9	1286.5	344.5

**Table 3:** Ablation study on different components of IRFNet. ↑ indicates higher is better; ↓ indicates lower is better; ✓ indicates the component is included; **Bold** values represent the best performance.

Basic	HFEM	CIOF	CAMO				COD10K				NC4K			
			M↓	$S_{\alpha }↑$	$E_{\phi }↑$	$F_{\beta }^{w}↑$	M↓	$S_{\alpha }↑$	$E_{\phi }↑$	$F_{\beta }^{w}↑$	M↓	$S_{\alpha }↑$	$E_{\phi }↑$	$F_{\beta }^{w}↑$
✓			0.075	0.805	0.839	0.734	0.032	0.829	0.883	0.727	0.048	0.845	0.885	0.777
✓	✓		0.062	0.843	0.893	0.788	0.025	0.864	0.922	0.778	0.040	0.869	0.914	0.811
✓		✓	0.055	0.858	0.914	0.817	0.022	0.881	0.939	0.809	0.035	0.882	0.927	0.833
✓	✓	✓	**0.047**	**0.869**	**0.920**	**0.830**	**0.020**	**0.884**	**0.942**	**0.817**	**0.032**	**0.888**	**0.932**	**0.845**

**Table 4:** Ablation study on different components of the HFEM. ↑ indicates higher is better; ↓ indicates lower is better; **Bold** values represent the best performance.

Method	CAMO				COD10K				NC4K			
	M↓	$S_{\alpha }↑$	$E_{\phi }↑$	$F_{\beta }^{w}↑$	M↓	$S_{\alpha }↑$	$E_{\phi }↑$	$F_{\beta }^{w}↑$	M↓	$S_{\alpha }↑$	$E_{\phi }↑$	$F_{\beta }^{w}↑$
*w*/*o* DCSA	0.054	0.859	0.914	0.818	0.021	0.881	0.939	0.812	0.033	0.887	**0.932**	0.844
*w*/*o* HMM	0.052	0.862	0.915	0.821	0.021	0.881	0.938	0.813	0.033	0.886	0.930	0.841
Ours	**0.047**	**0.869**	**0.920**	**0.830**	**0.020**	**0.884**	**0.942**	**0.817**	**0.032**	**0.888**	**0.932**	**0.845**

**Table 5:** Ablation study on different components of CIOF. ↑ indicates higher is better; ↓ indicates lower is better; **Bold** values represent the best performance.

Method	CAMO				COD10K				NC4K			
	M↓	$S_{\alpha }↑$	$E_{\phi }↑$	$F_{\beta }^{w}↑$	M↓	$S_{\alpha }↑$	$E_{\phi }↑$	$F_{\beta }^{w}↑$	M↓	$S_{\alpha }↑$	$E_{\phi }↑$	$F_{\beta }^{w}↑$
*w*/*o* GCAM	0.052	0.864	0.918	0.824	0.020	0.883	0.941	0.815	0.034	0.884	0.929	0.838
*w*/*o* IOM	0.059	0.850	0.901	0.796	0.025	0.864	0.924	0.779	0.040	0.870	0.916	0.811
Ours	**0.047**	**0.869**	**0.920**	**0.830**	**0.020**	**0.884**	**0.942**	**0.817**	**0.032**	**0.888**	**0.932**	**0.845**

**Table 6:** Ablation study on different backbone architectures. The best and second-best results are highlighted in **bold** and underline, respectively;↑ indicates higher is better; ↓ indicates lower is better.

Backbone	Efficiency		CAMO				COD10K				NC4K			
	**Params (M)**↓	**FLOPs (G)**↓	M↓	$S_{\alpha }↑$	$E_{\phi }↑$	$F_{\beta }^{w}↑$	M↓	$S_{\alpha }↑$	$E_{\phi }↑$	$F_{\beta }^{w}↑$	M↓	$S_{\alpha }↑$	$E_{\phi }↑$	$F_{\beta }^{w}↑$
ResNet-50	**25.99**	475.99	0.086	0.785	0.845	0.701	0.029	0.847	0.910	0.751	0.046	0.853	0.902	0.784
Res2Net-50	48.84	560.21	0.092	0.772	0.818	0.682	0.029	0.844	0.907	0.745	0.045	0.851	0.899	0.786
ConvNeXt-B	89.28	403.34	0.051	**0.869**	**0.922**	**0.835**	0.020	**0.884**	**0.944**	**0.819**	0.033	0.888	0.935	0.847
Swin-S	50.52	286.59	0.051	0.864	0.919	0.828	**0.019**	0.883	0.943	0.814	**0.032**	**0.891**	**0.937**	**0.850**
PVT-v2-B2	27.21	**203.42**	**0.047**	**0.869**	0.920	0.830	0.020	**0.884**	0.942	0.817	**0.032**	0.888	0.932	0.845

**Table 7:** Ablation study on different numbers of total iterations. ↑ indicates higher is better; ↓ indicates lower is better; **Bold** values represent the best performance.

#Iters	CAMO				COD10K				NC4K			
	M↓	$S_{\alpha }↑$	$E_{\phi }↑$	$F_{\beta }^{w}↑$	M↓	$S_{\alpha }↑$	$E_{\phi }↑$	$F_{\beta }^{w}↑$	M↓	$S_{\alpha }↑$	$E_{\phi }↑$	$F_{\beta }^{w}↑$
1	0.054	0.860	0.911	0.817	0.021	**0.884**	**0.942**	0.816	0.033	0.885	0.930	0.839
2	0.053	0.860	0.911	0.818	0.021	0.881	0.939	0.814	0.033	0.887	0.931	0.843
3	**0.047**	**0.869**	**0.920**	**0.830**	**0.020**	**0.884**	**0.942**	**0.817**	**0.032**	**0.888**	**0.932**	**0.845**
4	0.054	0.858	0.911	0.816	0.021	0.882	0.938	0.815	0.033	0.886	0.929	0.843
5	0.056	0.853	0.908	0.810	0.021	0.882	0.938	0.813	0.033	0.887	0.931	0.843

**Table 8:** Detailed performance metrics for each iteration in our optimal three-iteration model. ↑ indicates higher is better; ↓ indicates lower is better; **Bold** values represent the best performance.

Iters	CAMO				COD10K				NC4K			
	M↓	$S_{\alpha }↑$	$E_{\phi }↑$	$F_{\beta }^{w}↑$	M↓	$S_{\alpha }↑$	$E_{\phi }↑$	$F_{\beta }^{w}↑$	M↓	$S_{\alpha }↑$	$E_{\phi }↑$	$F_{\beta }^{w}↑$
1	0.053	0.859	0.912	0.805	0.025	0.861	0.924	0.771	0.038	0.872	0.920	0.813
2	0.048	**0.869**	**0.920**	0.829	**0.020**	0.882	0.940	0.812	0.033	0.885	0.930	0.840
3	**0.047**	**0.869**	**0.920**	**0.830**	**0.020**	**0.884**	**0.942**	**0.817**	**0.032**	**0.888**	**0.932**	**0.845**

**Table 9:** Ablation study on different supervision strategies. ↑ indicates higher is better; ↓ indicates lower is better; **Bold** values represent the best performance.

Method	CAMO				COD10K				NC4K			
	M↓	$S_{\alpha }↑$	$E_{\phi }↑$	$F_{\beta }^{w}↑$	M↓	$S_{\alpha }↑$	$E_{\phi }↑$	$F_{\beta }^{w}↑$	M↓	$S_{\alpha }↑$	$E_{\phi }↑$	$F_{\beta }^{w}↑$
Single Branch	0.055	0.863	0.908	0.815	0.021	0.886	0.935	0.813	0.034	0.890	0.927	0.839
Combined Branch	0.053	0.865	0.911	0.820	0.021	**0.888**	0.938	0.816	0.033	**0.892**	0.929	0.840
Ours	**0.047**	**0.869**	**0.920**	**0.830**	**0.020**	0.884	**0.942**	**0.817**	**0.032**	0.888	**0.932**	**0.845**

**Table 10:** Quantitative comparison of polyp segmentation performance. The best and second-best results are highlighted in **bold** and underline, respectively;↑ indicates higher is better; ↓ indicates lower is better.

Method	ETIS-LaribPolypDB				CVC-ColonDB				Kvasir				CVC-300				CVC-ClinicDB			
	M↓	$S_{\alpha }↑$	$E_{\phi }↑$	$F_{\beta }^{w}↑$	M↓	$S_{\alpha }↑$	$E_{\phi }↑$	$F_{\beta }^{w}↑$	M↓	$S_{\alpha }↑$	$E_{\phi }↑$	$F_{\beta }^{w}↑$	M↓	$S_{\alpha }↑$	$E_{\phi }↑$	$F_{\beta }^{w}↑$	M↓	$S_{\alpha }↑$	$E_{\phi }↑$	$F_{\beta }^{w}↑$
UNet [28]	0.036	0.684	0.643	0.366	0.059	0.710	0.691	0.491	0.055	0.858	0.881	0.794	0.022	0.843	0.847	0.684	0.019	0.889	0.913	0.811
UNet++ [29]	0.035	0.683	0.629	0.390	0.061	0.692	0.680	0.467	0.048	0.862	0.886	0.808	0.018	0.839	0.834	0.687	0.022	0.873	0.891	0.785
SFA [10]	0.109	0.557	0.532	0.231	0.094	0.629	0.661	0.366	0.075	0.782	0.834	0.670	0.065	0.640	0.644	0.341	0.042	0.793	0.840	0.647
PraNet [11]	0.031	0.794	0.808	0.600	0.043	0.820	0.847	0.699	0.030	0.915	0.944	0.885	0.010	0.925	0.950	0.843	0.009	0.936	0.963	0.896
CFANet [12]	**0.014**	0.845	0.881	0.693	0.039	0.835	0.869	0.728	0.023	0.924	0.956	0.903	0.008	**0.938**	0.962	**0.875**	**0.007**	**0.950**	**0.981**	0.924
Ours	**0.014**	**0.875**	**0.902**	**0.754**	**0.028**	**0.873**	**0.915**	**0.797**	**0.021**	**0.929**	**0.964**	**0.912**	**0.006**	0.935	**0.965**	0.871	**0.007**	0.947	0.977	**0.927**

## Data Availability

The training datasets are publicly available at https://github.com/DengPingFan/SINet, accessed on 1 March 2025. The test datasets can be found at https://drive.google.com/file/d/1QEGnP9O7HbN_2tH999O3HRIsErIVYalx/view, accessed on 1 March 2025 and https://drive.google.com/file/d/1kzpX_U3gbgO9MuwZIWTuRVpiB7V6yrAQ/view, accessed on 1 March 2025. All additional data used in this study, including images and source code, are available upon reasonable request from the corresponding author.
